# Challenges and opportunities of English as the medium of instruction in diploma midwifery programs in Bangladesh: a mixed-methods study

**DOI:** 10.1186/s12909-024-05499-8

**Published:** 2024-05-10

**Authors:** Anna Williams, Jennifer R. Stevens, Rondi Anderson, Malin Bogren

**Affiliations:** 1 Data, Design + Writing, Portland, OR USA; 2 Goodbirth Network, North Adams, USA MA; 3 Project HOPE, Washington DC, USA; 4 University of Gothenburg, Gothenburg, Sweden

**Keywords:** Midwifery, Education, English, “English for special purposes”, “English medium instruction”, Bangladesh

## Abstract

**Background:**

English is generally recognized as the international language of science and most research on evidence-based medicine is produced in English. While Bangla is the dominant language in Bangladesh, public midwifery degree programs use English as the medium of instruction (EMI). This enables faculty and student access to the latest evidence-based midwifery content, which is essential for provision of quality care later. Yet, it also poses a barrier, as limited English mastery among students and faculty limits both teaching and learning.

**Methods:**

This mixed-methods study investigates the challenges and opportunities associated with the implementation of EMI in the context of diploma midwifery education in Bangladesh. Surveys were sent to principals at 38 public midwifery education institutions, and 14 English instructors at those schools. Additionally, ten key informant interviews were held with select knowledgeable stakeholders with key themes identified.

**Results:**

Surveys found that English instructors are primarily guest lecturers, trained in general or business English, without a standardized curriculum or functional English language laboratories. Three themes were identified in the key informant interviews. First, in addition to students’ challenges with English, faculty mastery of English presented challenges as well. Second, language labs were poorly maintained, often non-functional, and lacked faculty. Third, an alternative education model, such as the English for Specific Purposes (ESP) curriculum,  has potential to strengthen English competencies within midwifery schools.

**Conclusions:**

ESP, which teaches English for application in a specific discipline, is one option available in Bangladesh for midwifery education. Native language instruction and the middle ground of multilingualism are also useful options. Although a major undertaking, investing in an ESP model and translation of technical midwifery content into relevant mother tongues may provide faster and more complete learning. In addition, a tiered system of requirements for English competencies tied to higher levels of midwifery education could build bridges to students to help them access global evidence-based care resources. Higher levels might emphasize English more heavily, while the diploma level would follow a multilingualism approach, teach using an ESP curriculum, and have complementary emphasis on the mother tongue.

## Introduction

As the international language of science, English holds an important position in the education of healthcare professionals. Globally, most scientific papers are published in English. In many non-native English-speaking countries, English is used as the language of instruction in higher education [1]. The dominant status held by the English language in the sciences is largely considered to increase global access to scientific information by unifying the scientific community under a single lingua franca [2].

In Bangladesh, where the mother tongue is Bangla and midwifery diploma programs are taught in English, knowledge of English facilitates student and instructor access to global, continuously updated evidence-based practice guidance. This includes basic and scientific texts, media-based instructional materials (including on life-saving skills), professional journals, and proceedings of medical conferences. Many of these resources are available for free online, which can be particularly useful in healthcare settings that have not integrated evidence-based practice.

In addition to opportunity though, English instruction also creates several challenges. Weak student and faculty English competency may impede midwifery education quality in Bangladesh. Globally, literature has linked limited instructor competency in the language of instruction with reduced depth, nuance, and accuracy in conveying subject matter content [3]. This can lead to the perpetuation of patterns of care in misalignment with global evidence. In addition, students’ native language proficiency in their topic of study can decline when instruction is in English, limiting native language communication between colleagues on the job later on [4, 5].

In this paper, we examine the current status of English language instruction within public diploma midwifery programs in Bangladesh. Midwifery students are not required to demonstrate a certain skill level in English to enter the program. However, they are provided with English classes in the program. Midwifery course materials are in English, while—for ease and practicality—teaching aids and verbal classroom instruction are provided in Bangla. Following graduation, midwifery students must pass a national licensing exam given in English to practice. Upon passing, some new midwives are deployed as public employees and are posted to sub-district health facilities where English is not used by either providers or clients. Others will seek employment as part of non-governmental organization (NGO) projects where English competency can be of value for interacting with global communities, and for participating in NGO-specific on-the-job learning opportunities. The mix of both challenge and opportunity in this context is complex.

Our analysis examines the reasons for the identified English competency gaps within midwifery programs, and potential solutions. We synthesize the findings and discuss solutions in the context of the global literature. Finally, we present a set of viable options for strengthening English competencies among midwifery faculty and students to enable better quality teaching and greater learning comprehension among students.

## Methods

### Study design

We employed a mixed-methods study design [6] in order to assess the quality of English instruction within education programs, and options for its improvement. Data collection consisted of two surveys of education institutes, a web-search of available English programs in Bangladesh, and key informant interviews. Both surveys followed a structured questionnaire with a combination of open- and closed-ended questions and were designed by the authors. One survey targeted the 38 institute principals and the other targeted 14 of the institutes’ 38 English instructors (those for whom contact information was shared). The web-search focused on generating a list of available English programs in Bangladesh that had viable models that could be tapped into to strengthen English competencies among midwifery faculty and students. Key informant interviews were unstructured and intended to substantiate and deepen understanding of the survey and web-search findings.

### Context

No minimum requirements exist for students’ English competencies upon entry into midwifery diploma programs. Students enter directly from higher secondary school (12th standard) and complete the midwifery program over a period of three years. Most students come from modest economic backgrounds having completed their primary and secondary education in Bangla. While English instruction is part of students’ secondary education, skill attainment is low, and assessment standards are not in place to ensure student mastery. To join the program, midwifery students are required to pass a multi-subject entrance exam that includes a component on English competency. However, as no minimum English standard must be met, the exam does not screen out potential midwifery students. Scoring, for instance, is not broken down by subject. This makes it possible to answer zero questions correctly in up to three of the subjects, including English, and pass the exam.

### Processes/data collection

Prior to the first survey, principals were contacted by UNFPA with information about the survey and all provided verbal consent to participate. The survey of principals collected general information about the resources available for English instruction at the institutes. It was a nine-item questionnaire with a mix of Yes/No, multiple choice and write-in questions. Specific measures of interest were whether and how many English instructors the institutes had, instructors’ hiring criteria, whether institutes had language labs and if they were in use, and principals’ views on the need for English courses and their ideal mode of delivery (e.g., in-person, online, or a combination). This survey also gathered contact information of institute English instructors. These measures were chosen as they were intended to provide a high-level picture of institutes’ English resources such as faculty availability and qualifications, and use of language labs. To ensure questions were appropriately framed, a pilot test was conducted with two institute principals and small adjustments were subsequently made. Responses were shared via an electronic form sent by email and were used to inform the second survey as well as the key informant interviews. Of the 38 principals, 36 completed the survey.

The second survey, targeting English instructors, gathered information on instructors’ type of employment (e.g., institute faculty or adjunct lecturers); length of employment; student academic focus (e.g., midwifery or nursing); hours of English instruction provided as part of the midwifery diploma program; whether a standard English curriculum was used and if it was tailored toward the healthcare profession; use of digital content in teaching; education and experience in English teaching; and their views on student barriers to learning English. These measures were chosen to provide a basic criterion for assessing quality of English instruction, materials and resources available to students. For instance, instructors’ status as faculty would indicate a stronger degree of integration and belonging to the institute midwifery program than a guest lecturer status which allows for part time instruction with little job security. In addition, use of a standard, professionally developed English curriculum and integration of digital content into classroom learning would be indicative of higher quality than learning materials developed informally by instructors themselves without use of listening content by native speakers in classrooms. The survey was piloted with two English instructors. Based on their feedback, minor adjustments were made to one question, and it was determined that responses were best gathered by phone due to instructors’ limited internet access. Of the 14 instructors contacted, 11 were reached and provided survey responses by phone.

The web-search gathered information on available English language instruction programs for adults in Bangladesh, and the viability of tapping into any of them to improve English competency among midwifery students and faculty. Keywords *Bangladesh* + *English courses*, *English training*, *English classes*, *study English* and *learn English* were typed into Google’s search platform. Eleven English language instruction programs were identified. Following this, each program was contacted either by phone or email and further detail about the program’s offerings was collected.

Unstructured key informant interviews were carried out with select knowledgeable individuals to substantiate and enhance the credibility of the survey and web-search findings. Three in-country expert English language instructors and four managers of English language teaching programs were interviewed. In addition, interviews were held with three national-level stakeholders knowledgeable about work to make functional technologically advanced English language laboratories that had been installed at many of the training institutes. Question prompts included queries such as, ‘In your experience, what are the major barriers to Bangla-medium educated students studying in English at the university level?’, ‘What effective methods or curricula are you aware of for improving student English to an appropriate competency level for successful learning in English?’, and, ‘What options do you see for the language lab/s being used, either in their originally intended capacity or otherwise?’

### Data analysis

All data were analyzed by the lead researcher. Survey data were entered into a master Excel file and grouped descriptively to highlight trends and outliers, and ultimately enable a clear description of the structure and basic quality attributes (e.g., instructors’ education, hours of English instruction, and curriculum development resources used). Web-search findings were compiled in a second Excel file with columns distinguishing whether they taught general English (often aimed at preparing students for international standard exams), Business English, or English for Specific Purposes (ESP). This enabled separation of standalone English courses taught by individual instructors as part of vocational or academic programs of study in other fields, and programs with an exclusive focus on English language acquisition. Key informant interviews were summarized in a standard notes format using Word. An inductive process of content analysis was carried out, in which content categories were identified and structured to create coherent meaning [7]. From this, the key overall findings and larger themes that grew from the initial survey and web-search results were drawn out.

## Results

The surveys (Tables 1 and 2) found that English instructors are primarily long-term male guest lecturers employed at each institute for more than two years. All principal respondents indicated that there is a need for English instruction—18 of the 19 reported that this is best done through a combination of in-person and computer-based instruction. Ten institutes reported that they have an English language lab, but none were used as such. The other institutes did not have language labs. The reported reasons for the labs not being in use were a lack of trained staff to operate them and some components of the technology not being installed or working properly. The findings from the instructors’ survey indicated that English instructors typically develop their own learning materials and teach general English without tailoring content to healthcare contexts. Only two mentioned using a standard textbook to guide their instruction and one described consulting a range of English textbooks to develop learning content. None reported using online or other digital tools for language instruction in their classrooms. Most instructors had an advanced degree (i.e., master’s degree) in English, and seven had received training in teaching English. Interviews with instructors also revealed that they themselves did not have mastery of English, as communication barriers in speaking over the phone appeared consistently across 10 of the 11 instructor respondents.

**Table 1:** Survey results from midwifery education institute principals

Response rate (N = 38)	Institutes (N = 19)		
	Has a language lab	Language lab used	Preferred teaching approach
Responded (19, 50%) --- No response (19, 50%)	Yes (10, 53%) No (9, 47%) --- No response (0, 0%)	Yes (2, 11%) No (17, 89%) --- No response (0, 0%)	In-person and on computer (18, 95%) In-person, no computer (1, 5%) --- No response (0, 0%)

**Table 2:** Survey results from midwifery education institute instructors

Response rate (N = 27)*	Instructors (N = 15)				Instructional content	
	Has English degree	Has training in teaching English	Employment status		Develops own materials versus uses existing curriculum	Curriculum tailored to students working in healthcare contexts
Responded (15, 19%) --- No response (12, 44%)	Yes (9, 60%) No (6, 40%) --- No response (0, 0%)	Yes (7, 47%) No (8, 53%) --- No response (0, 0%)	Guest lecturer (13, 87%) Staff (0, 0%) ---- No response (2, 13%)		Develop own materials (5, 33%) Use existing curriculum (2, 13%) --- No response (8, 53%)	Yes (0, 0%) No (7, 100%) --- No response (8, 53%)

*Twenty-seven English instructors were contacted

The web-search and related follow up interviews found that most English instruction programs (10 out of the 11) were designed for teaching general English and/or business English. The majority were offered through private entities aiming to reach individuals intending to study abroad, access employment that required English, or improve their ability to navigate business endeavors in English. One program, developed by the British Council, had flexibility to tailor its structure and some of its content to the needs of midwifery students. However, this was limited in that a significant portion of the content that would be used was developed for global audiences and thus not tailored to a Bangladeshi audience or to any specific discipline. One of the university English programs offered a promising ESP model tailored to midwifery students. It was designed by BRAC University’s Institute of Language for the university’s private midwifery training program.

Three themes emerged from the other key informant interviews (Table 3). The first was that, in addition to students’ challenges with English, faculty mastery of English presented challenges as well. Of the 34 faculty members intending to participate in the 2019–2020 cohort for the Dalarna master’s degree, half did not pass the prerequisite English exam. Ultimately, simultaneous English-Bangla translation was necessary for close to half of the faculty to enable their participation in the master’s program. English language limitations also precluded one faculty member from participating in an international PhD program in midwifery.

The second theme highlighted the language labs’ lack of usability. The language labs consisted of computers, an interactive whiteboard, audio-visual equipment, and associated software to allow for individualized direct interactions between teacher and student. However, due to the lack of appropriately trained staff to manage, care for and use the language lab equipment, the investment required to make the labs functional appeared to outweigh the learning advantages doing so would provide. Interviews revealed that work was being done, supported by a donor agency, on just one language lab, to explore whether it could be made functional. The work was described as costly and challenging, and required purchasing a software license from abroad, thus likely being impractical to apply to the other labs and sustain over multiple years.

The third theme was around the ESP curriculum model. The program developers had employed evidence-informed thinking to develop the ESP learning content and consulted student midwives on their learning preferences. Due to the student input, at least 80% of the content was designed to directly relate to the practice of midwifery in Bangladesh, while the remaining 10–20% references globally relevant content. This balance was struck based on students’ expressed interest in having some exposure to English usage outside of Bangladesh for their personal interest. For conversation practice, the modules integrated realistic scenarios of midwives interacting with doctors, nurses and patients. Also built into written activities were exercises where students were prompted to describe relevant health topics they are concurrently studying in their health, science or clinical classes. Given the midwifery students’ educational backgrounds and intended placements in rural parts of Bangladesh, an ESP curriculum model appeared to be the most beneficial existing program to pursue tapping into to strengthen English competencies within midwifery programs. This was because the content would likely be more accessible to students than a general English course by having vocabulary, activities and examples directly relevant to the midwifery profession.

**Table 3:** Themes and their descriptions from key informant interviews

Theme	Description	Respondents (N = 10)
Faculty mastery of English	Limited faculty mastery of English became apparent when faculty required translation support to participate in international master’s program in sexual and reproductive health.	Shared by five respondents, which included all with knowledge of faculty English competencies.
Language lab usability	Interviews revealed that institutes did not have staff trained in using the lab technology for teaching, or for maintaining and troubleshooting the equipment.	Shared by all three interviewees knowledgeable about the language labs.
Benefits of English for Specific Purposes curriculum	The English for Specific Purposes curriculum appeared to be an accessible and practical model for students due to its custom design for Bangladeshi midwifery students, and the applicability of the examples and practice exercises to public facility clinical settings where midwives are deployed following graduation.	The two English for Specific Purposes curriculum managers described the positive results of the curriculum for English language improvement; managers of the other programs did not describe student results.

## Discussion

The study findings demonstrate key weaknesses in the current model of English instruction taught in public midwifery programs. Notably, the quantitative findings revealed that some English instructors do not have training in teaching English, and none used standard curricula or online resources to structure and enhance their classroom content. In addition, weak mastery of English among midwifery faculty was identified in the qualitative data, which calls into question faculty’s ability to fully understand and accurately convey content from English learning materials. Global literature indicates that this is not a unique situation. Many healthcare faculty and students in low-resource settings, in fact, are faced with delivering and acquiring knowledge in a language they have not sufficiently mastered [8]. As a significant barrier to knowledge and skill acquisition for evidence-based care, this requires more attention from global midwifery educators [9].

Also holding back students’ English development is the finding from both the quantitative and qualitative data that none of the high-tech language labs were being used as intended. This indicates a misalignment with the investment against the reality of the resources at the institutes to use them. While setting up the costly language labs appears to have been a large investment with little to no return, it does demonstrate that strengthening English language instruction in post-secondary public education settings is a priority that the Bangladesh government is willing to invest in. However, scaling up access to an ESP curriculum model tailored to future midwifery practitioners in Bangladesh may be a more worthwhile investment than language labs [10]. 

The ESP approach teaches English for application in a specific discipline. It does this by using vocabulary, examples, demonstrations, scenarios and practice activities that are directly related to the context and professions those studying English live and work (or are preparing to work) in. One way ESP has been described, attributed to Hutchinson and Waters (1987), is, “ESP should properly be seen not as any particular language product but as an approach to language teaching in which all decisions as to content and method are based on the learner’s reason for learning” [11]. It is proposed by linguistic education researchers as a viable model for strengthening language mastery and subject matter comprehension in EMI university contexts [12].

Though it did not arise as a finding, reviewing the literature highlighted that Bangla language instruction may be an additional, potentially viable option. Linguistic research has long shown that students learn more thoroughly and efficiently in their mother tongue [12]. Another perhaps more desirable option may be multilingualism, which entails recognizing native languages as complementary in EMI classrooms, and using them through verbal instruction and supplemental course materials. Kirkpatrick, a leading scholar of EMI in Asia, suggests that multilingualism be formally integrated into EMI university settings [13]. This approach is supported by evidence showing that the amount of native language support students need for optimal learning is inversely proportional to their degree of English proficiency [14].

Ultimately, despite the language related learning limitations identified in this study, and the opportunities presented by native language and multilingualism approaches, there remains a fundamental need for members of the midwifery profession in Bangladesh to use up-to-date guidance on evidence-based midwifery care [11]. Doing that currently requires English language competence. Perhaps a tiered system of requirements for English competencies that are tied to diploma, Bachelor’s, Master’s and PhD midwifery programs could build bridges for more advanced students to access global resources. Higher academic levels might emphasize English more heavily, while the diploma level could follow a multilingualism approach—teaching using an ESP curriculum and integrating Bangla strategically to support optimal knowledge acquisition for future practice in rural facilities. Ideally, scores on a standard English competency exam would be used to assess students’ language competencies prior to entrance in English-based programs and that this would require more stringent English skill development prior to entering a midwifery program.

### Methodological considerations

One of the limitations of this study is that it relied on self-reports and observation, rather than tested language and subject matter competencies. Its strengths though are in the relatively large number of education institutes that participated in the study, and the breadth of knowledge about faculty and student subject matter expertise among study co-authors. It was recognized that the lead researcher might be biased toward pre-determined perceptions of English competencies being a barrier to teaching and learning held by the lead institution (UNFPA). It was also recognized that due to the inherent power imbalance between researcher and participants, the manner of gathering data and engaging with stakeholders may contribute to confirmation bias, with respondents primarily sharing what they anticipated the researcher wished to hear (e.g., that English needed strengthening and the lead agency should take action to support the strengthening). The researcher thus engaged with participants independently of UNFPA and employed reflexivity by designing and carrying out the surveys to remotely collect standard data from institutes, as well as casting a wide net across institutes to increase broad representation. In addition, while institutes were informed that the surveys were gathering information about the English instruction within the institutes, no information was shared about potential new support to institutes. Finally, the researcher validated and gathered further details on the relevant information identified in the surveys through key informant interviews, which were held with stakeholders independent of UNFPA.

## Conclusion

Adapting and scaling up the existing ESP modules found in this study, and integrating Bangla where it can enhance subject-matter learning, may be a useful way to help midwifery students and faculty improve their knowledge, skills, and critical thinking related to the field of midwifery. Given the educational backgrounds and likely work locations of most midwives in Bangladesh and many other LMICs, practitioners may want to consider investing in more opportunities for local midwives to teach and learn in their mother tongue. This type of investment would ideally be paired with a tiered system in which more advanced English competencies are required at higher-levels of education to ensure integration of global, evidence-based approaches into local standards of care.

Declarations.

## Data Availability

The datasets used and analyzed during the current study are available from the corresponding author upon reasonable request.

## Abbreviations

BRAC: Bangladesh Rehabilitation Assistance Committee
EMI: English medium instruction
ESP: English for Specific Purposes
LMICs: Low- and Middle-Income Countries
MOHFW: Ministry of Health and Family Welfare
UNFPA: United Nations Population Fund
